# E-cigarette use and associated factors among adults aged 18–44 years in China: Findings from an online survey

**DOI:** 10.18332/tid/191994

**Published:** 2024-09-07

**Authors:** Xiaochen Yang, Xiaoyue Zhang, Lanchao Zhang, Wangnan Cao, Chengqian Zhang, Xiangsu Wang, Shiyuan Wang, Chun Chang

**Affiliations:** 1 School of Public Health, Peking University, Beijing, China; 2 Jiangsu Provincial Center for Disease Control and Prevention, Nanjing, China; 3 School of Public Health, Southeast University, Nanjing, China

**Keywords:** e-cigarettes, prevalence, perception, cross-sectional survey

## Abstract

**INTRODUCTION:**

There needs to be more up-to-date evidence on the prevalence of e-cigarette use among Chinese adults. This study aims to investigate the prevalence and associated factors of e-cigarette use among adults aged 18–44 years in China.

**METHODS:**

Cross-sectional design and convenience sampling were used. The data for this study were obtained from an online survey conducted in mainland China from April to May 2023. The target population was adults aged 18–44 years. Descriptive analysis was employed to report the prevalence of e-cigarette use, while adjusted multivariable logistic regression was performed to examine the association between e-cigarette use and related factors.

**RESULTS:**

A total of 4256 participants were included in this study; 12.9% were current e-cigarette users, 5.9% were frequent users, and 7.0% were occasional users. The descriptive analysis results indicated that males and cigarette users had a higher prevalence of e-cigarette use. Multivariable analysis showed that e-cigarette use was significantly associated with female gender (AOR=0.76, 95% CI: 0.60–0.96), those aged 25–34 years with monthly income 6000–8999 CNY (AOR=2.01; 95% CI: 1.18–3.41), those aged 25–34 years with monthly income ≥9000 CNY (AOR=2.20; 95% CI: 1.26–3.82), college or undergraduate degree (AOR=1.91; 95% CI: 1.22–3.00), urban residence (AOR=1.72; 95% CI: 1.34–2.20), being a current smoker (AOR=3.32; 95% CI: 2.64–4.16), perception of harm (AOR=0.66; 95% CI: 0.60–0.73), and perception of benefit (AOR=2.31; 95% CI: 2.04–2.61).

**CONCLUSIONS:**

The prevalence of current e-cigarette use among adults in China was 12.9% within our sample. In addition to sociodemographic factors, individuals with a higher perception of the harm associated with e-cigarettes were less likely to engage in e-cigarette consumption. Conversely, individuals who perceive the ‘benefits’ of e-cigarettes more favorably use them. Targeted interventions, such as health education, are recommended to help adults develop a correct understanding of e-cigarettes and lower the prevalence of e-cigarette use.

## INTRODUCTION

Tobacco use remains a major public health challenge in China, as the prevalence of current smoking was 26.6% in 2018^1^. The consumption of tobacco increases the risk of chronic diseases, such as chronic obstructive pulmonary disease, cancer, and diabetes^2,3^. Evidence suggests that the risk of developing at least one chronic disease is approximately 3.86 times higher among heavy smokers than among non-smokers^4^. The Chinese government aims to reduce smoking prevalence among people aged ≥15 years to 20% by 2030, as outlined in the Healthy China 2030 Action Plan, recognizing the urgency of tobacco control^5^.

Electronic cigarettes (e-cigarettes) have become increasingly popular worldwide^6^. According to the China Adult Tobacco Survey, the proportion of adults aged ≥15 years who used e-cigarettes increased from 3.1% in 2015 to 5.0% in 2018, with a higher prevalence among males (9.3%)^1,7^. The increasing prevalence of e-cigarettes has prompted concerns among the general public regarding their potential health effects. For example, the consumption of sweet-flavored e-cigarettes could increase the likelihood of starting smoking, leading to dual use or greater nicotine addiction^8^.

The WHO 8th report on the global tobacco epidemic^9^, published in 2021, provided new data on e-cigarettes, emphasizing the harmful effects of e-cigarettes and the need for effective regulation. A study analyzing e-cigarette policies across 68 countries revealed that approximately a third of countries have no regulations specific to e-cigarettes^10^. In 2022, the Chinese government enacted the Regulation on the Administration Measures of E-cigarettes, which integrated e-cigarettes into the tobacco product regulatory framework, in line with the global trend of e-cigarette regulation^11^.

Previous studies suggested that sociodemographic factors such as age, income and education were associated with e-cigarette use patterns and preferences^12,13^. Evidence from some surveys conducted in China found that young adults, high-income groups, individuals with higher level of education, and urban residents were more likely to use e-cigarettes^14,15^. With the updated regulations for e-cigarettes in China^16^, including restrictions on sales channels and increased public awareness, the demographic characteristics of e-cigarette users may change. For example, Zhao et al.^14^ found that the use of e-cigarettes increased in both urban and rural areas from 2015 to 2016 as annual household income rose. However, individuals with low household income also showed a high prevalence of e-cigarette use in both areas from 2018 to 2019. Additionally, perceptions and attitudes towards e-cigarettes, including perceived harm or benefit, impact the decision to use them or abstain^17^.

Several surveys on e-cigarette use have been conducted in China, including the China Chronic Disease and Nutrition Surveillance Program^14^ and the China Tobacco-Free Cities Program in 2017–2018^15^. Due to the rapid economic development and increasing popularity of e-cigarettes, up-to-date data are necessary. According to the 2018 China Adult Tobacco Survey Report^1^, those aged 15–44 years had the highest percentage of e-cigarette users, which is consistent with previous studies^18,19^. Thus, this study aims to estimate the prevalence of e-cigarette use among adults aged 18–44 years in China and identify the factors associated with e-cigarette use.

## METHODS

### Study design and sample

This cross-sectional study was conducted online in mainland China from April to May 2023. We used the convenience sampling method to select participants, but we also applied probability sampling principles to determine the sample size^20^. The criteria of participation were: 1) adults aged 18–44 years, and 2) able to independently complete the questionnaire. This study was reviewed and approved by the Biomedical Ethics Committee of Peking University (Approval number: IRB00001052-23091), and all participants provided informed consent.

We anticipated that the e-cigarette use rate was 9.5%, with α (significance level) set at 0.05 and a presumed standard error of 10%, a sample size of 3659 was required. To avoid biased sample selection, we conducted sampling according to the age and sex proportion of the national population in 2020. Considering the additional bias of the convenience sampling method, we expanded the sample size to >4000 participants. Data were collected through online anonymous questionnaires. We utilized the Wenjuanxing platform, a large online survey company in China, for participant recruitment in this study. Various studies have utilized the Wenjuanxing platform for data collection, indicating its widespread use^21,22^. We utilized the sample services of Wenjuanxing, where the sample pool was selected based on research requirements to conduct surveys, ensuring the sample was representative.

Initially, a pre-survey was distributed to assess potential interest in participation in the survey. Subsequently, the formal questionnaire was distributed to those interested in participating. Before participants answered the questionnaire, they were presented with an informed consent page. If participants agreed to participate in the study, they could click on the ‘Agree’ option and proceed to answer the questionnaire. The Wenjuanxing platform’s self-check function identified unqualified responses, such as those with random responses or excessively short completion times (<300 s). Finally, the submitted questionnaires were reviewed by researchers at Peking University, and any questionnaires with identified issues (such as discrepancies between birthdate and age) were promptly rechecked on the platform.

### Measures

*E-cigarette and cigarette use*

We measured the smoking status based on the following questions: ‘Do you currently smoke? (Including traditional cigarettes and e-cigarettes)’. There were three response options: ‘Yes, currently smoking’, ‘Used to smoke, but not anymore’, and ‘Never smoked’. Participants who chose the first two options were asked a second question: ‘Which do you smoke, cigarettes or e-cigarettes?’. Response options were: ‘cigarettes,’ ‘e-cigarettes,’ or ‘both’. Current e-cigarette users were then asked: ‘Do you use it regularly or occasionally?’. Response options were: ‘frequent use’ or ‘occasional use’. Additionally, we defined current e-cigarette users and former e-cigarette users as ‘ever e-cigarette users’.

*Sociodemographic characteristics*

The main demographic characteristics in this study include age, gender, marital status, education level, residence, and income. Age was categorized into age groups of 18–24, 25–34, and 35–44 years. Education level was divided into three categories: high school or lower, college or undergraduate degree, and Master’s or higher. Marital status included unmarried, married, and widowed/divorced/separated. Monthly income in CNY (1000 Chinese Yuan about US$140) was divided into four categories: <3000 , 3000–5999, 6000–8999, and ≥9000. Residence was defined as either living in urban areas, or living in rural towns or villages.

*Perception of harm and benefit regarding e-cigarette use*

The perception of the benefit and harm of e-cigarette use was measured using 12 questions. The design of these questions was based on previous studies on e-cigarette perception^17,19^ and tailored to our study objectives. Each question had five response options (strongly disagree, disagree, neutral, agree, strongly agree), and scores were assigned from 0 to 4. All participants in this study were required to respond (Cronbach’s alpha=0.86). In addition, to calculate participants’ perception rates of each question in the study, we categorized scores from 3 to 4 as ‘Agree’ and scores from 0 to 2 as ‘Disagree/Neutral’.

The following six questions were used to measure the perception of harm: 1) ‘Do you agree that e-cigarettes are harmful to your health?’; 2) ‘Do you agree that e-cigarette users are more likely to smoke cigarettes?’; 3) ‘Do you agree that using e-cigarettes can have a negative impact on your life?’; 4) ‘Do you agree that e-cigarette users are more likely to get addicted?’; 5) ‘Do you agree that e-cigarette users are more likely to have cardiovascular and lung diseases?’; and 6) ‘Do you agree that e-cigarette users are more likely to be anxious?’.

The variable of perception of benefit was also measured by six questions: 1) ‘Do you agree that using e-cigarettes can help to refresh yourself?’; 2) ‘Do you agree that using e-cigarettes can bring pleasure and enjoyment?’; 3) ‘Do you agree that smoking e-cigarettes helps you concentrate better?’; 4) ‘Do you agree that using e-cigarettes is fashionable?’; 5) ‘Do you agree that using e-cigarettes will not bother others?’; and 6) ‘Do you agree that e-cigarettes can facilitate socialization?’.

### Statistical analysis

We assessed the characteristics of participants stratified by e-cigarette use status. The characteristics of participants according to e-cigarette use status were compared using chi-squared tests for categorical variables. We calculated perception rates for each question on e-cigarette use and conducted chi-squared tests to compare differences between subgroups by e-cigarette use status. To assess the effectiveness of the questions in measuring the perception of harm and benefit of using e-cigarettes and to calculate the scores for each indicator more scientifically, we employed principal component analysis (PCA) with varimax rotation by the R package *psych*. This analysis method linearly combines the original variables to create new composite variables and achieves dimensionality reduction. Additionally, we used a scree plot to determine the number of principal components, and component loadings to reflect each question’s contribution to the principal components. Analysis of variance (ANOVA) or t-tests were used to compare differences in principal component scores between different subgroups. The principal component scores from PCA, representing perceptions of e-cigarette harm and benefit, were included in subsequent multivariable logistic regression models to evaluate factors associated with current and ever use of e-cigarettes in the study population. Both models adjusted for age, gender, marital status, education level, residence, income, current cigarette use status, and perceptions of using e-cigarettes. All tests were two-sided with a significance level of p<0.05. Data analysis and visualizations were conducted using R-4.3.0 (R Foundation for Statistical Computing, Vienna, Austria).

## RESULTS

### Basic characteristics

This study ultimately included 4256 participants, and the basic characteristics are presented in Table 1. Among the participants, 51.6% were male, 42.3% were aged 25–34 years, 64.6% were married, and 63.0% resided in urban areas. Additionally, 32.3% were cigarette smokers, 12.9% were current e-cigarette users (5.9% frequent users, 7% occasional users), and 16.7% were ever e-cigarette users.

**Table 1:** Demographic characteristics of the study participants by e-cigarette use status, among Chinese adults aged 18–44 years, 2023 (N=4256)

*Characteristics*	*All n (%)*	*Current e-cigarette user*				*Ever e-cigarette user*			
		*Yes n (%)*	*Non (%)*	*χ^2^*	*p*	*Yes n (%)*	*Non (%)*	*χ^2^*	*p*
**Age** (years)				60.67	<0.01			65.22	<0.01
18–24	864 (20.3)	56 (6.5)	808 (93.5)			83 (9.6)	781 (90.4)		
25–34	1801 (42.3)	306 (17.0)	1495 (83.0)			389 (21.6)	1412 (78.4)		
35–44	1591 (37.4)	186 (11.7)	1405 (88.3)			240 (15.1)	1351 (84.9)		
**Gender**				102.86	<0.01			73.22	<0.01
Male	2196 (51.6)	394 (17.9)	1802 (82.1)			472 (21.5)	1724 (78.5)		
Female	2060 (48.4)	154 (7.5)	1906 (92.5)			240 (11.7)	1820 (88.3)		
**Marital status**				36.69	<0.01			38.70	<0.01
Never married	1438 (33.8)	126 (8.8)	1312 (91.2)			176 (12.2)	1262 (87.8)		
Married	2748 (64.6)	417 (15.2)	2331 (84.8)			531 (19.3)	2217 (80.7)		
Widowed/divorced/separated	70 (1.6)	5 (7.1)	65 (92.9)			5 (7.1)	65 (92.9)		
**Education level**				23.29	<0.01			17.59	<0.01
High school or lower	465 (10.9)	27 (5.8)	438 (94.2)			46 (9.9)	419 (90.1)		
College or undergraduate degree	3471 (81.6)	478 (13.8)	2993 (86.2)			608 (17.5)	2863 (82.5)		
Master’s or higher	320 (7.5)	43 (13.4)	277 (86.6)			58 (18.1)	262 (81.9)		
**Residence**				64.02	<0.01			69.69	<0.01
Rural	1576 (37.0)	118 (7.5)	1458 (92.5)			165 (10.5)	1411 (89.5)		
Urban	2680 (63.0)	430 (16.0)	2250 (84.0)			547 (20.4)	2133 (79.6)		
**Monthly income** (CNY)				151.21	<0.01			143.86	<0.01
<3000	734 (17.2)	29 (4.0)	705 (96.0)			46 (6.3)	688 (93.7)		
3000–5999	1140 (26.8)	84 (7.4)	1056 (92.6)			130 (11.4)	1010 (88.6)		
6000–8999	1325 (31.1)	221 (16.7)	1104 (83.3)			276 (20.8)	1049 (79.2)		
≥9000	1057 (24.8)	214 (20.2)	843 (79.8)			260 (24.6)	797 (75.4)		
**Cigarette smoker**				357.82	<0.01			151.76	<0.01
Yes	1376 (32.3)	371 (27.0)	1005 (73.0)			371 (27.0)	1005 (73.0)		
No	2880 (67.7)	177 (6.1)	2703 (93.9)			341 (11.8)	2539 (88.2)		

CNY: 1000 Chinese Yuan about US$140.

### Perception rates and scores of harm and benefit of e-cigarette use

Regarding the perception of harm of e-cigarette use, over half of all respondents believed ‘e-cigarettes are harmful to your health’ and ‘using e-cigarettes can have a negative impact on your life’. These perceptions differed by e-cigarette use status (Table 2). Current and ever users were less likely than non-users to perceive e-cigarettes as harmful. For the perception of benefit, more than 40% of all participants believed using e-cigarettes ‘can help to refresh yourself’, ‘can bring pleasure and enjoyment’, and ‘will not bother others’. Only 15% of the participants reported that ‘e-cigarettes can facilitate socialization’. These beliefs also differed by e-cigarette use status. Both current and ever users were more likely than non-users to perceive e-cigarettes as having varying degrees of benefits (Table 2).

**Table 2:** Perception rates of harm and benefit of e-cigarettes in the study participants by e-cigarette use status, among Chinese adults aged 18–44 years, 2023 (N=4256)

*Items*	*All %*	*Current e-cigarette user*				*Ever e-cigarette user*			
		*Yes %*	*No %*	*χ^2^*	*p*	*Yes %*	*No %*	*χ^2^*	*p*
**Perception of harm of e-cigarettes**									
Harmful to your health	53.36	37.04	55.77	66.53	<0.01	37.22	56.60	88.73	<0.01
Users are more likely to smoke cigarettes	37.08	25.55	38.78	35.27	<0.01	25.84	39.33	45.68	<0.01
Using them can have a negative impact on your life	50.21	33.76	52.64	67.35	<0.01	35.53	53.16	72.98	<0.01
Users are more likely to get addicted	44.45	27.19	47.01	75.13	<0.01	26.54	48.05	110.20	<0.01
Users are more likely to have cardiovascular and lung diseases	46.55	32.66	48.60	48.08	<0.01	34.55	48.96	48.87	<0.01
Users are more likely to be anxious	44.13	27.92	46.52	66.25	<0.01	28.93	47.18	79.32	<0.01
**Perception of benefit of e-cigarette use**									
Can help to refresh yourself	48.05	78.47	43.55	231.74	<0.01	75.98	42.44	265.93	<0.01
Can bring pleasure and enjoyment	41.99	73.72	37.30	258.57	<0.01	71.07	36.15	295.41	<0.01
Can help to concentrate better	27.75	54.01	23.87	214.93	<0.01	52.67	22.74	263.34	<0.01
Fashionable	35.62	54.20	32.87	93.73	<0.01	50.84	32.56	85.60	<0.01
Will not bother others	42.27	60.40	39.59	83.89	<0.01	58.15	39.08	87.54	<0.01
Can facilitate socialization	15.06	29.56	12.92	102.09	<0.01	27.39	12.58	100.40	<0.01

The data in the table represent the percentage of individuals who agree with the statement. For analysis purposes, responses were dichotomized (score of 3 or 4=agree, and score of 0, 1, or 2=disagree/neutral).

The scree plot (Supplementary file Figure 1) of the principal component analysis indicated that two principal components (with eigenvalues greater than 1) can explained most variations among the 12 questions. The factor loadings indicated that the first principal component primarily measured perception of harm of e-cigarette use, while the second principal component primarily measured perception of benefit of e-cigarette use (Supplementary file Table 1). Table 3 presents the principal component scores among various subgroups. Both current and ever e-cigarette users exhibited lower scores in principal component-1 (perception of harm) compared with their counterparts, while scoring higher in principal component-2 (perception of benefit).

**Table 3:** Principal component scores of the study participants, among Chinese adults aged 18–44 years, 2023 (N=4256)

*Characteristics*	*Principal component 1: perception of harm*			*Principal component 2: perception of benefit*		
	*Mean ± SD*	*F/t*	*p*	*Mean ± SD*	*F/t*	*p*
**Age** (years)		58.03	<0.01		101.30	<0.01
18–24	0.30 ± 0.91			-0.42 ± 0.97		
25–34	-0.01 ± 1.01			0.07 ± 1.00		
35–44	-0.15 ± 1.00			0.15 ± 0.95		
**Gender**		4.60	<0.01		9.06	<0.01
Male	-0.07 ± 1.00			0.13 ± 0.99		
Female	0.07 ± 0.99			-0.14 ± 0.99		
**Marital status**		103.20	<0.01		146.80	<0.01
Never married	0.23 ± 0.93			-0.28 ± 0.97		
Married	-0.12 ± 1.02			0.15 ± 0.99		
Widowed/divorced/separated	0.00 ± 0.95			-0.10 ± 0.96		
**Education level**		4.53	0.03		4.28	0.04
High school or lower	-0.10 ± 1.01			-0.10 ± 0.99		
College or undergraduate degree	0.01 ± 1.00			0.01 ± 1.01		
Master’s or higher	0.04 ± 0.99			0.03 ± 0.95		
**Residence**		3.63	<0.01		8.01	<0.01
Rural	0.07 ± 0.99			-0.17 ± 0.98		
Urban	-0.04 ± 1.01			0.10 ± 1.00		
**Monthly income** (CNY)		72.11	<0.01		199.40	<0.01
<3000	0.29 ± 0.90			-0.38 ± 0.94		
3000–5999	0.04 ± 0.99			-0.11 ± 0.97		
6000–8999	-0.14 ± 1.01			0.13 ± 1.00		
≥9000	-0.06 ± 1.02			0.22 ± 0.98		
**Cigarette smoker**		9.74	<0.01		17.21	<0.01
Yes	-0.22 ± 1.02			0.36 ± 0.93		
No	0.10 ± 0.97			-0.17 ± 0.98		
**Current e-cigarette user**		8.97	<0.01		21.29	<0.01
Yes	-0.35 ± 0.97			0.70 ± 0.80		
No	0.05 ± 0.99			-0.10 ± 0.98		
**Ever e-cigarette user**		9.78	<0.01		22.15	<0.01
Yes	-0.32 ± 0.96			0.64 ± 0.81		
No	0.07 ± 0.99			-0.13 ± 0.99		

CNY: 1000 Chinese Yuan about US$140.

### Current e-cigarette use

Table 1 shows the prevalence of current e-cigarette use in the overall study population and subgroups. The prevalence of current e-cigarette use was higher among males (17.9%) compared to females (7.5%). The age group with the highest proportion of e-cigarette use was 25–34 years, with a prevalence of 17.0%. The prevalence of current e-cigarette use increased with income, with the highest percentage (20.2%) observed among individuals with a monthly income ≥9000 CNY. Additionally, being married and having a college or undergraduate degree, as well as a Master’s degree or higher, were associated with a higher percentage of e-cigarette use. In terms of cigarette use status, current cigarette smokers were more likely to use e-cigarettes (27.0%) compared to non-smokers (6.1%).

**Figure 1:**
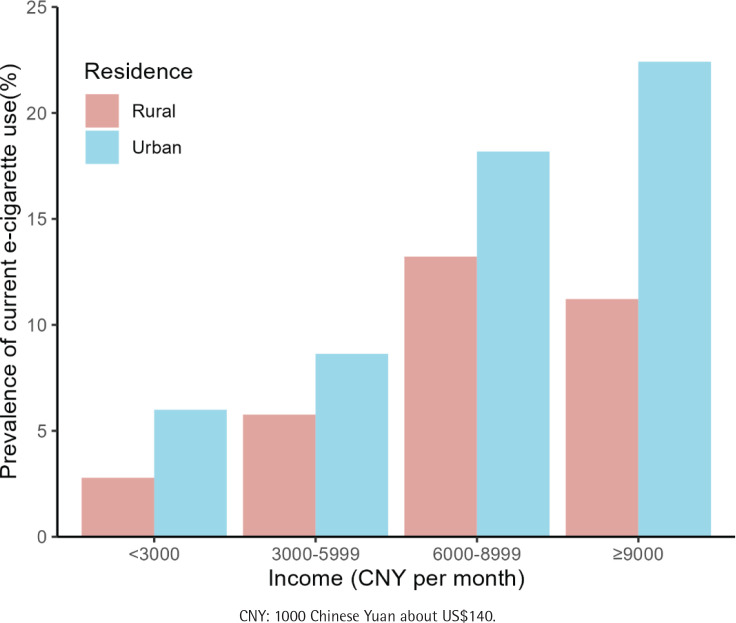
Prevalence of current e-cigarette use by residence and income, among Chinese adults aged 18-44 years, 2023 (N=4256)

Figure 1 illustrates the prevalence of current e-cigarette use based on residence and income. Among the urban population, the prevalence of current e-cigarette use increased with higher income. However, in rural areas, the prevalence of current e-cigarette use initially increased with income and then decreased.

**Figure 2:**
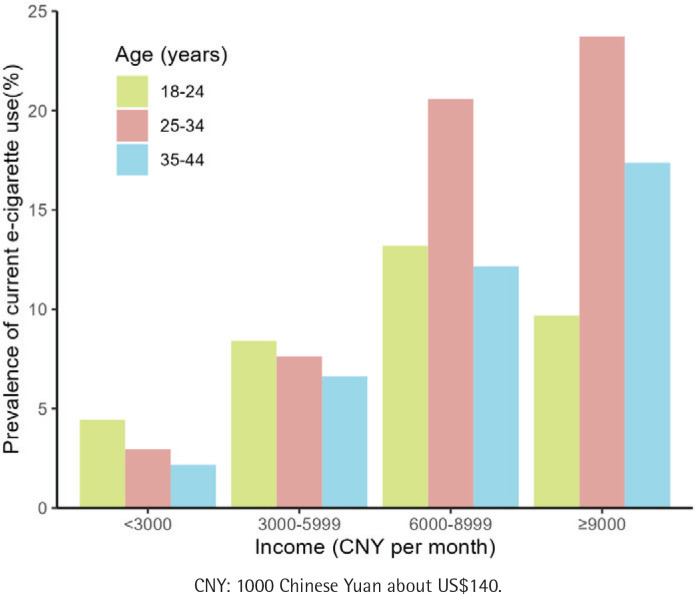
Prevalence of current e-cigarette use by age and income, among Chinese adults aged 18-44 years, 2023 (N=4256)

Figure 2 displays the prevalence of current e-cigarette use stratified by age and income. In the low-income group (<3000 and 3000–5999 CNY per month), the age group of 18–24 years had the highest e-cigarette use rate, while in the high-income group (6000–8999 and ≥9000 CNY per month), the age group of 25–34 years had the highest e-cigarette use rate.

**Figure 3:**
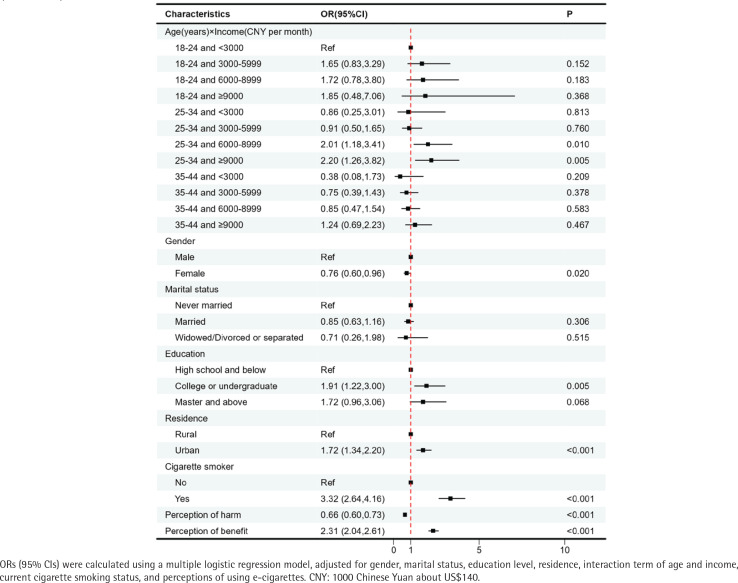
Factors associated with current e-cigarette use, among Chinese adults aged 18-44 years, 2023 (N=4256)

We included age and income as two independent variables in the multivariable logistic model. The results showed that the odds ratios (ORs) for the age groups of 25–34 and 35–44 years were <1, indicating that older age groups were less likely to use e-cigarettes (Supplementary file Figure 2). Furthermore, we replaced the variables of age and income with their interaction term and conducted the multivariable analysis (Table 3). Participants aged 25–34 years with a monthly income of 6000–8999 CNY (AOR=2.01; 95% CI: 1.18–3.41) and those aged 25–34 years with a monthly income of ≥9000 CNY (AOR=2.20; 95% CI: 1.26–3.82) had higher ORs of current e-cigarette use compared to the reference group (aged 18–24 years with a monthly income of <3000 CNY). Females had a lower likelihood of using e-cigarettes compared to males (AOR=0.76; 95% CI: 0.60–0.96). The association of marital status with e-cigarette use was no longer significant. Individuals with an education level of college or undergraduate degree were more likely to use e-cigarettes than those with a high school or lower (AOR=1.91; 95% CI: 1.22–3.00). Additionally, living in urban areas (AOR=1.72; 95% CI: 1.34–2.20) and being current cigarette smokers (AOR=3.32; 95% CI: 2.64–4.16) were associated with a higher likelihood of current e-cigarette use. It is worth noting that there was a statistically significant association between perceptions of e-cigarettes and current e-cigarette use. Individuals with higher levels of perceived harm regarding e-cigarettes were less likely to use them (AOR=0.66; 95% CI: 0.60–0.73), while those who perceived higher benefit from e-cigarette use were more likely to use them (AOR=2.31; 95% CI: 2.04–2.61).

### Ever e-cigarette use

The subgroup differences in the prevalence of ever e-cigarette use were similar to those observed in current e-cigarette use (Table 1). For example, the prevalence of ever e-cigarette use was higher among males compared to females (21.5% vs 11.7%), and the highest prevalence of ever e-cigarette use was observed in those aged 25–34 years (21.6%). In the multivariable logistic regression analysis, we first constructed a model including age and income. The results showed that older age group (35–44 years) had lower odds of ever using e-cigarettes, whereas higher income was associated with higher odds of ever using e-cigarettes (Supplementary file Figure 3). Furthermore, we included the interaction term between age and income in the model, and the results indicated that e-cigarette ever use was found to be significantly associated with gender, the interaction of age and income, residence, current cigarette use, and perception of harm and benefit (Supplementary file Figure 4).

## DISCUSSION

This study provides up-to-date evidence for e-cigarette use among adults in China, showing that the prevalence of current, frequent, and occasional e-cigarette use within our sample was 12.9%, 5.9%, and 7.0%, respectively. The prevalence was higher than those reported in previous studies^14,23^, possibly due to the focus of this study on the age group of 18–44 years. A similar study targeting Chinese young adults (aged 18–29 years) indicated an e-cigarette use rate of 24.45%, with only 2.34% reporting frequent use (≥20 times/week)^24^. Findings reported in another Chinese survey showed that young adults aged 15–24 years (8.5% and 4.1%) and 25–44 years (7.8% and 1.3%) had a higher prevalence of both ever and current e-cigarette use compared with other age groups^18^.

A complex relationship between age, income, and e-cigarette use was observed in this study. E-cigarette use was more prevalent among young adults, which is consistent with previous studies^12,25^. The age group of 25–34 years had the highest rate of e-cigarette use in our findings. The multivariable analysis indicated that individuals in the age group of 25–35 years with higher income levels had a higher likelihood of using e-cigarettes. This could be attributed to the strong correlation between age and income, and e-cigarette use may be influenced by both age and income rather than a single factor alone. The price of e-cigarettes is generally higher than that of cigarettes^26^. Individual’s purchasing power, which is related to their income, may affect their decision to use e-cigarettes.

Individuals with a higher level of education were more likely to be current e-cigarette users in our study, but this link was not observed among ever e-cigarette users. The study conducted by Adkison et al.^27^ also found similar results. E-cigarettes are often considered a less harmful alternative to cigarettes and can be used to quit smoking^6,28^. Individuals with a higher level of education were more aware of the harmful effects of smoking and were more positive about using e-cigarettes^13^. Considering that ever users include individuals who have quit e-cigarettes, unmeasured confounding factors such as changes in e-cigarette regulations may explain this result. From the residence perspective, there was a higher prevalence of e-cigarette use in urban areas compared to rural areas. This difference may be related to the fact that e-cigarettes are sold only in authorized stores in China, which makes them more accessible to urban residents^11^.

This study also showed a relatively high prevalence of dual use of cigarettes and e-cigarettes. Coleman et al.^29^ found that 44.3% of the participants maintained dual smoking status during the study period. The prevalence of e-cigarette use was higher among cigarette smokers. For cigarette smokers, e-cigarettes play a role in substituting conventional cigarettes, fulfilling similar needs with fewer health risks^28^. Previous evidence revealed that quitting cigarette use was among the primary reasons for using e-cigarettes^30^. The effectiveness of e-cigarettes for cigarette cessation remains controversial. Recent studies indicated that e-cigarettes were detrimental to health despite the difficulty in observing the harm in a short period^31^. Transiting to e-cigarette users may reduce the health risks associated with cigarette use, but becoming a dual user could increase the health hazards^32^.

The perception of harm and benefit of e-cigarette use differed between users and non-users. Previous studies suggested that many users believe e-cigarettes are less harmful than cigarettes and could provide benefits such as stress relief and pleasure for themselves, which was consistent with our findings^33,34^. Personal perceptions often precede the formation of behaviors and are closely associated with subsequent changes in behavior. Perceptions of e-cigarettes play a role in the adoption of use behaviors. A study conducted by Sanders-Jackson et al.^35^ suggested that the public, especially young adults, had inaccurate knowledge of e-cigarettes. With increasing evidence of the health risks associated with e-cigarettes, public perceptions of the harm of e-cigarettes also increase^36^.

Despite increased regulation of e-cigarettes by the Chinese government and society over the years, the current situation of e-cigarettes still requires further efforts. China’s regulatory authorities implemented e-cigarette management measures on 1 May 2022, and mandatory national standards for e-cigarettes on 1 October 2022^16^. These policies prohibit the sale of e-cigarettes to individuals under the age of 18 years and restrict the flavors and ingredients that can be added to e-cigarette products^11^. This represents a new phase in the management of e-cigarettes in China. Perhaps the effects of these measures will be gradually revealed.

### Limitations

This study has some limitations. First, we used convenience sampling instead of random sampling, which may have introduced selection bias. However, we tried to minimize this bias by increasing the sample size and matching it to the national age-sex distribution. Second, the participants self-reported their e-cigarette use and associated factors, which may have led to recall errors. Thirdly, as this study relied on an online survey, it is crucial to acknowledge the potential for residual confounding caused by unmeasured variables that could impact e-cigarette use. Finally, the findings may have limited generalizability to other countries or regions with different socio-cultural contexts and regulatory environments. Longitudinal studies are warranted to monitor e-cigarette use and perception in the population and to evaluate the long-term effects of relevant policies and interventions.

## CONCLUSIONS

This study provides timely and important evidence for understanding the current status of e-cigarette perceptions and use among Chinese population. The high prevalence of e-cigarette use among young people in China needs more attention. Factors associated with e-cigarette use include gender, age, income, education level, smoking status, and perceptions of using e-cigarettes. Future research and investigations on e-cigarettes are crucial to understanding the trends of e-cigarette use within the Chinese population. It is recommended to promote the perceptions of e-cigarettes and provide health education to high-risk e-cigarette user groups.

## Data Availability

The data supporting this research are available from the authors on reasonable request.
